# The effect of skin fatty acids on *Staphylococcus aureus*

**DOI:** 10.1007/s00203-014-1048-1

**Published:** 2014-10-18

**Authors:** Yvonne Neumann, Knut Ohlsen, Stefanie Donat, Susanne Engelmann, Harald Kusch, Dirk Albrecht, Michael Cartron, Alexander Hurd, Simon J. Foster

**Affiliations:** 1 Department of Molecular Biology and Biotechnology, The Krebs Institute, University of Sheffield, Firth Court, Western Bank, Sheffield, S10 2TN UK; 2 Institute of Molecular and Clinical Immunology, Otto-von-Guericke-University Magdeburg, Leipziger Str. 44, 39120 Magdeburg, Germany; 3 Research Group of Systems-Oriented Immunology and Inflammation Research, Department of Immune Control, Helmholtz Centre for Infection Research, Braunschweig, Germany; 4 Institute for Molecular Infection Biology, University of Würzburg, Würzburg, Germany; 5 Institute for Microbiology, Ernst-Moritz-Arndt-University, Greifswald, Germany; 6 Institute for Microbiology, Technical University Braunschweig, Braunschweig, Germany; 7 Research Group for Microbial Proteomics, Helmholtz Centre for Infection Research, Braunschweig, Germany; 8 Institute for Microbiology and Genetics, University of Göttingen, Göttingen, Germany

**Keywords:** *S. aureus*, Skin fatty acid, C-6-H, Resistance

## Abstract

*Staphylococcus aureus* is a commensal of the human nose and skin. Human skin fatty acids, in particular *cis*-6-hexadecenoic acid (C-6-H), have high antistaphylococcal activity and can inhibit virulence determinant production. Here, we show that sub-MIC levels of C-6-H result in induction of increased resistance. The mechanism(s) of C-6-H activity was investigated by combined transcriptome and proteome analyses. Proteome analysis demonstrated a pleiotropic effect of C-6-H on virulence determinant production. In response to C-6-H, transcriptomics revealed altered expression of over 500 genes, involved in many aspects of virulence and cellular physiology. The expression of toxins (*hla, hlb,* *hlgBC*) was reduced, whereas that of host defence evasion components (*cap, sspAB, katA*) was increased. In particular, members of the SaeRS regulon had highly reduced expression, and the use of specific mutants revealed that the effect on toxin production is likely mediated via SaeRS.

**Electronic supplementary material:**

The online version of this article (doi:10.1007/s00203-014-1048-1) contains supplementary material, which is available to authorized users.

## Introduction

The Gram-positive bacterium *Staphylococcus aureus* is able to survive as a commensal organism in the anterior nares and on human skin. A third of the human population are nasal carriers and two-thirds are intermittent carriers, forming a large reservoir for potential infection (Peacock et al. 2001). As a pathogen, *S. aureus* is highly adaptable, with an alarming spread of antibiotic resistance. This limits the range of effective therapies able to combat this organism. *S. aureus* is able to cause a wide range of diseases, from minor skin infections to severe systemic disease (such as bacteraemia, septic arthritis and endocarditis). Also, in the hospital environment, *S. aureus* is responsible for many infections associated with surgical wounds and catheters. The ability to inhabit so many niches with such a range of infectious sequelae is due to a large repertoire of virulence determinants.

The human body has many innate defence mechanisms to prevent infection by invading microbes. Physical barriers (human skin and mucosa) prevent pathogens from ingress. The human skin is composed of tightly bound epithelial cells and covered by a highly cross-linked layer of keratin and is therefore normally impenetrable to bacteria (Proksch et al. 2008). Additionally, the skin produces antimicrobial peptides as well as skin fatty acids which are crucial for host defence (Ong et al. 2002; Niyousaba and Ogawa 2005). Several fatty acids have been isolated from human skin, which have strong antimicrobial activity (Miller et al. 1988; Wille and Kydonieus 2003). The antibacterial activity of unsaturated fatty acids has been well known for many years (Kabara et al. 1972; Knapp and Melly 1986; Shin et al. 2007), the most effective antistaphylococcal skin fatty acid being *cis*-6-hexadecanoic acid (C-6-H, sapienic acid, C16:1Δ6) (Takigawa et al. 2005; Wille and Kydonieus 2003). As well as being antibacterial, C-6-H also has the ability to inhibit virulence determinant production and the induction of antibiotic resistance mechanisms (Clarke et al. 2007; Projan et al. 1994; Schlievert et al. 1992; Takigawa et al. 2005; Kenny et al. 2009). In fact, in murine models of *S. aureus* infection, C-6-H has shown to be an effective treatment. Thus, it is important to understand how C-6-H mediates its effects and the response of *S. aureus* to such assault. A surface protein, IsdA, has been shown to be involved in resistance of *S. aureus* to C-6-H by rendering the cells more hydrophilic (Clarke et al. 2007). Also, wall teichoic acids are required to prevent susceptibility to C-6-H (Kohler et al. 2009).

In order to define bacterial components important in resistance to C-6-H and how its effect on virulence determinant expression is mediated, a global study of gene expression and protein profile analysis in response to C-6-H was carried out.

## Materials and methods

### Bacterial strains and culture conditions

Bacterial strains used in this study are listed in Table 1 and were grown in iron-limited tryptic soy broth (TSB^−Fe^) (Oxoid), Chelex-100 (Sigma Aldrich), with the addition of 20 µM 2,2′-dipyridyl (Baldassarri et al. 2001). Antibiotics used were erythromycin (5 µg/ml), lincomycin (25 µg/ml) or tetracycline (5 µg/ml) where appropriate. Cultures were grown at 37 °C and inoculated with an overnight culture to an optical density at 600 nm (OD_600_) of 0.05 into TSB^−Fe^, followed by incubation with agitation at 37 °C. Bacterial growth was monitored by measuring the OD_600_.

**Table 1:** Strains used in this study

Strain	Genotype/markers	Reference
SH1000	Functional *rsbU* ^*+*^ derivative of 8325-4	Horsburgh et al. (2001)
Newman	High level of clumping factor	Duthie and Lorent (1952)
JLA371	SH1000 *hla*∷*lacZ hla* ^+^ (Ery^R^)	Horsburgh et al. (2001)
SJF1293	*saeS*∷Tn*551* (SH1000) (Ery^R^)	Needham et al. (2004)
SJF1295	*saeR*∷Tn*551* (SH1000) (Ery^R^)	Needham et al. (2004)
Reynolds CP5	Serotype 5 prototype strain (CP5)	Karakawa and Vann (1982)
Reynolds (CP^−^)	Capsule-negative mutant of Reynolds (CP5) (Ery^R^)	Watts et al. (2005)
KC046	*mrgA*∷*lacZ* (pAZ106) (Ery^R^)	Cosgrove (unpublished)

### Bacterial killing assays

Bacteria were grown to an OD_600_ of approximately 0.6 in TSB^−Fe^. Cells from 10 ml of culture were harvested by centrifugation for 10 min at 5,000×*g* and 4 °C. The cell pellet was washed twice in sterile dH_2_O by resuspension and centrifugation as above. OD_600_ was measured, and cell suspension was adjusted to OD_600_ of 1.0. Cells were incubated at 37 °C with *cis*-6-hexadecanoic acid (C-6-H; Matreya) and cfu determined at intervals by plating onto TSB^−Fe^ agar. For C-6-H pre-exposure experiments, bacteria were grown to an OD_600_ of 0.5 in TSB^−Fe^ and 8 µg/ml C-6-H was added, prior to continued incubation, with shaking, for 2 h by 37 °C. Cells were then harvested, washed and exposed to C-6-H in the killing assay, as above.

### Transcriptional analysis

Total RNA was isolated from cultures (OD_600_ of 0.5), prior to, 10 and 60 min after the addition of 10 µg/ml C-6-H. “RNAprotect Bacteria Reagent” (QIAGEN, Hilden, Germany) was added to 8 ml culture and incubated for 5 min, and cells were harvested by centrifugation (5,000×*g* for 10 min at 4 °C) and resuspended in 1 ml RLT buffer (Qiagen) including 10 µg/ml β-mercaptoethanol. Cells were lysed using a Fast Prep shaker (BIO 101 Savant, Haarlem, The Netherlands) for 3× 40 s at a speed of 6.5 units. RNA was isolated using an “RNeasy Mini Kit 250” from QIAGEN. RNA quantity was measured using a NanoDrop 1000 spectrophotometer and the quality checked by analysis with an Agilent 2100 Bioanalyzer (Agilent Technologies, Palo Alto, CA, USA). Reverse transcription and fluorescent labelling reactions were performed using 10 µg total RNA, random hexamer primers mix (Invitrogen), SuperScript III™ Reverse Transcriptase (Invitrogen) and incubation for 1 h at 50 °C. The cDNA was labelled with Cy3- and Cy5-dyed d’CTPs (Amersham) according to the manufacturer’s instructions (Scienion, Berlin, Germany).

RNA obtained from three independent biological experiments was utilised, and a dye switch experiment was performed to minimise errors based on the differential dye bleaching or incorporation absorption of Cy3 and Cy5 during the RT reaction. The microarray hybridization and washing of the slides were carried out as recommended by the manufacturer (Scienion, Berlin). Microarray hybridization was at 49 °C for 48 h. The microarrays (Scienion) contained the full genome of *S. aureus* N315. Each slide contained PCR products of 2,334 genes in duplicate copies of each open reading frame (ORF) and multiple controls. Slides were scanned using a Genepix 4000B laser scanner (Axon Instruments Inc., Union City, CA, USA), and the individual signal intensity was analysed using Acuity 4.0 software, according to the manufacture’s instructions. Briefly, data were normalised to the mean ratio of means of all features, and all experiments were normalised to each other. Standard deviations and mean values of gene expression ratios based on the two spot replicates on each microarray and three different hybridisation experiments were calculated. Significant changes in gene expression were identified by a mean ratio <0.5 or >2.0 and a *p* value <0.05.

### Real-time polymerase chain reaction (RT-qPCR)

RNA was isolated as described for the transcriptional analysis. Two microgram of RNA was reverse-transcribed to cDNA as above. Master mixes were prepared according to the manufacturer’s instructions, using oligonucleotides specific for target genes listed in Table 2. SYBR Green (SensiMix*Plus* SYBR, Quantace, London, UK) was used as a fluorescent nucleic acid dye. RT-qPCR was performed in a Mx3000P Real-Time PCR System (Stratagene), and the following temperature profile was used for amplification. The initial denaturation was at 95 °C for 10 min, and templates were amplified by 40 cycles at 95 °C for 30 s and 54 °C for 1 min. A final step, 1 min at 95 °C, 30 s at 54 °C and 30 s 95 °C was used. A dissociation curve was generated to ensure amplification of a single product and absence of primer dimmers (Nolan et al. 2006). Three reference genes (*gyrB*, *yneR* and *ysxC*) were used which showed no significant change in expression on microarrays at all times and under all conditions. For calculation of the relative levels of gene expression, only *gyrB* was used as the endogenous reference gene.

**Table 2:** Oligonucleotides used for RT-PCR analysis

Oligonucleotides	Sequence (5′–3′)
gyrB_QF	ATCACAGCATTTGGTACAGG
gyrB_QR	CGATAGAAGAATGTTAATAACAATGTT
ysxC_QF	GCAGTAAAAGAAGAACAATATCC
ysxC_QR	GGGTTGCTGTGATGTACG
asp23_QF	AAACAACAAGAACAAAATCAAGAG
asp23_QR	ACCACCTTCAACAGATACACC
hprT_QF	TGTAAGGAATTGGGAGCAC
hprT_QR	ACTTCACCAGTTGACTCAG
sceD_QF	TCGCATCATCATTAGCAGTAG
sceD_QR	GTGATAAGTAAACCCTTCATAGTC
saeS_QF	GTATTGGCATTATACCAGAACTAC
saeS_QR	GCGAGTTCATTAGCTATATATAAGC
saeR_QF	CCAAGGGAACTCGTTTTACG
saeR_QR	CATAGGGACTTCGTGACCAT
lytS_QF	AAAGTTGAAAGAAGTGCATACTAAAGAAG
lytS_QR	TGTACCGACGATAGAACCATG
lytR_QF	ATTAGGAGCTAAGATTCAAAAGATG
lytR_QR	TTGACTGCTTGTTCAATACG
lrgA_QF	GCATCAAAACCAGCACACTT
lrgA_QR	TGATGCAGGCATAGGAATTG
lrgB_QF	TATTTGGTGTGGCCTTCCTC
lrgB_QR	AAACAGATTGTTGCCGGTTC
PhoP_QF	TCGGGTATTAGGTTTAGAATTAGG
PhoP_QR	GGTAATATCATCGTCAATCTCTTC
PhoR_QF	AATCCGTCCCATTCAAGAAGTTAC
PhoR_QR	AGGCGTCGTGCTAAATCATTG
butA_QF	CGTCTGAAGGTATTACTGTGAATG
butA_QR	TGAGAAACTCTGCCCAAAGC
agrB_QF	TCTGACGGTACTTATGTGAC
agrB_QR	CCAGTGCTATTAGTTCCACTG
lytM_QF	GCTATACATTCGTAGATGCTCAAG
lytM_QR	CTCGCTGTGTAGTCATTGTTATC
hla_QF	ATGATAGAGATTCTTGGAACCC
hla_QR	AATAACTGTAGCGAAGTCTGG
katA_QF	ACGAGATCCTAGAACAAATATGAG
katA_QR	GTATGTGTGAGAACCGAACC
clfA_QF	AATGATTCAAGTAGCGTTAGTG
clfA_QR	TTCGTTGTCGTAGTAGTAGC
sarA_QF	GAGTTGTTATCAATGGTCACTTATGC
sarA_QR	CAGTTCTTTCATCATGCTCATTACG
cidA_QF	CTACTACTACAACTAGGAATCATC
cidA_QR	TTTAGCGTAATTTCGGAAGC
mrgA_QF	AGTACAATCTAACATACCCACAATTTCTTG
mrgA_QR	GAGTGCTAATTCAGTTACGACTTTCTTG
rsbU_QF	GAAATCGTTAAAGGCTTTGGTTATAG
rsbU_QR	GCTCATTGTGCCATCGTTATG
spa_QF	GCAAACCATGCAGATGCTAA
spa_QR	AACGCTGCACCTAAGGCTAA

### Preparation of protein extracts

For the preparation of extracellular protein extracts, cytoplasmic protein extracts, ionically bound proteins and membrane proteins, bacteria were grown in TSB^−Fe^ (1 l) to exponential phase (OD_600_ of 1.0) and stationary phase (16 h).

Cells were harvested by centrifugation (9,000×*g* for 10 min), and extracellular proteins from the supernatant were precipitated using 100 % w/v fresh TCA (10 % w/v final volume) and incubated on ice for 30 min. Proteins were harvested by centrifugation (9,000×*g* for 5 min), and the pellet was washed 5 times with 100 % v/v acetone and air-dried for 1.5 h. The precipitated proteins were then washed twice with 100 % v/v ethanol, once with 70 % v/v ethanol and finally with 100 % v/v ethanol, prior to air-drying the pellet overnight at room temperature (RT). Proteins were dissolved in urea solution (8 M urea, 2 M thiourea), with incubation at RT for 30 min. In order to remove insoluble proteins, the suspension was centrifuged for 5 min at 20,000×*g*. The protein concentration was determined for each sample and adjusted to 200 µg protein.

For the isolation of the cytoplasmic proteins, harvested cells were broken by FastPrep as above and the suspension then centrifuged at 20,000×*g* for 10 min at 4 °C. The ensuing supernatant was centrifuged at 150,000×*g* for 2 h at 4 °C, using an ultracentrifuge (Optima™ LE-80 K, Beckman, USA). The supernatant was then centrifuged as above, and the cytoplasmic proteins were then precipitated using 10 % w/v TCA and prepared as described above (for supernatant proteins).

### Analytical and preparative 2D-PAGE

Two-dimensional polyacrylamide gel electrophoresis (2D-PAGE) was performed by using the immobilized pH gradient (IPG) technique as described previously (Bernhardt et al. 1999). First, protein samples were separated on linear IPG strips (ImmobilineTM DryStrips, GE Healthcare, Little Chalfont, UK). For extracellular and ionically bound protein samples, strips in the pH range 3–10 were used, and for cytoplasmic protein samples, the pH range 4–7 was used. 2D gels were loaded with 200 µg protein extract, and the resulting gels were fixed with 50 % v/v ethanol and 3 % v/v acetic acid, for 30 min. Afterwards, 2D gels were stained with SYPRO^®^-Ruby and fixed with 10 % v/v methanol and 7 % v/v acetic acid. The stained gels were finally scanned using a Typhoon 9400 Variable Mode Imager (Amersham Biosciences, Freiburg, Germany). For protein identification by matrix-assisted laser desorption ionisation-time-of-flight mass spectrometry (MALDI-TOF-MS), SYPRO stained protein spots were cut from gels using a spot cutter (Proteome Work™) with a picker head of 2 mm and transferred into 96-well microtiter plates. The proteins were digested with trypsin, and subsequent spotting of peptide solutions onto MALDI targets was performed automatically by using an Ettan Spot Handling Workstation (GE Healthcare, Little Chalfont, UK) using a standard protocol as previously described (Eymann et al. 2004). Actual analyses of spotted peptide solutions were performed as previously described (Wolf et al. 2008).

## Results and discussion

### Induced resistance of *S. aureus* to C-6-H

**Fig. 1:**
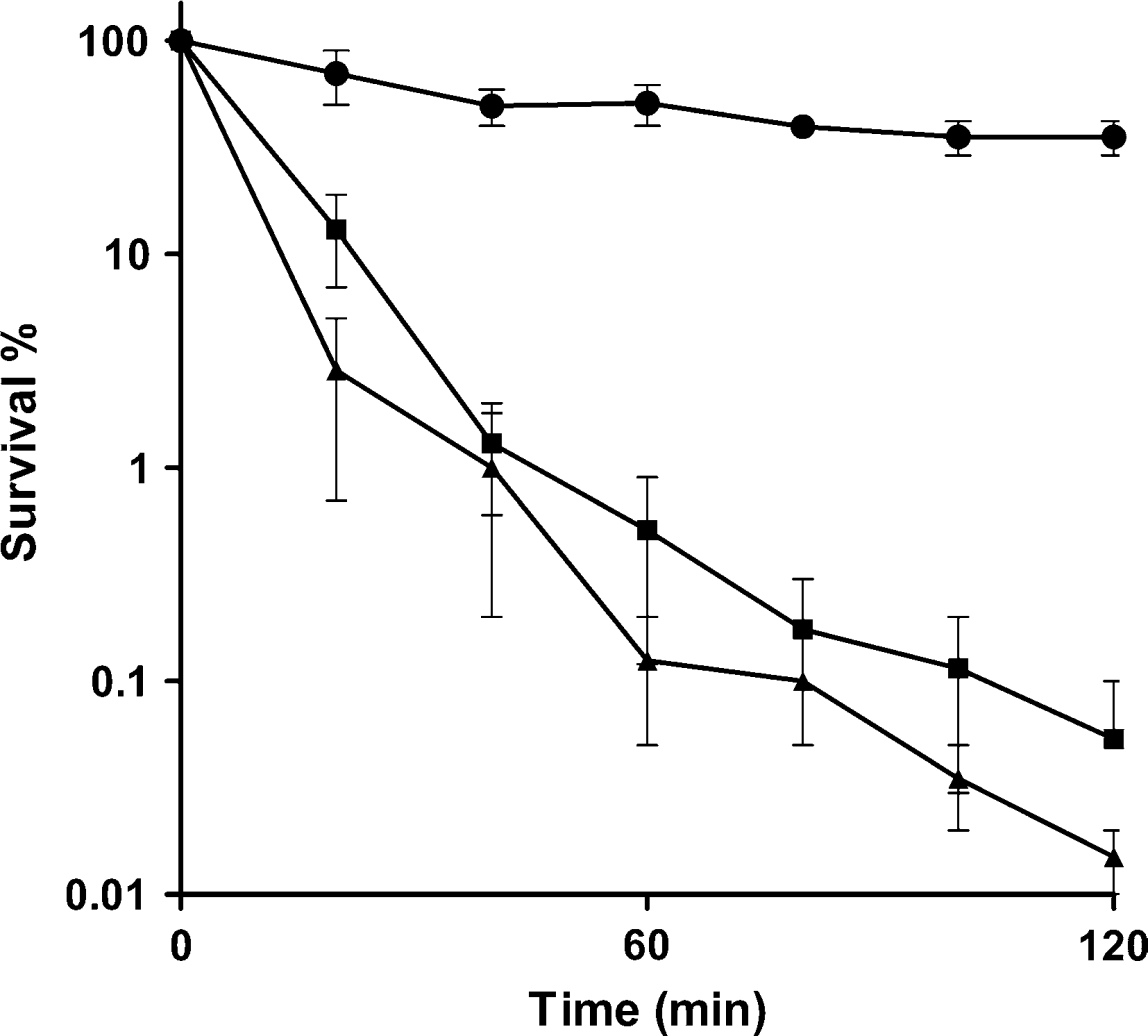
Bactericidal effect of C-6-H on strain SH1000 wt. SH1000 was grown in TSB^−Fe^ until OD_600_ 0.5–0.6. Cells were harvested, washed with dH_2_O and challenged with 0 (*filled circle*), 10 (*filled square*) or 20 (*filled triangle*) µg/ml C-6-H over 2 h. Samples were taken, and cfu was determined over time. Samples were plated in triplicate, and each experiment was repeated twice. *Error bars* indicate the standard error of the mean

**Fig. 2:**
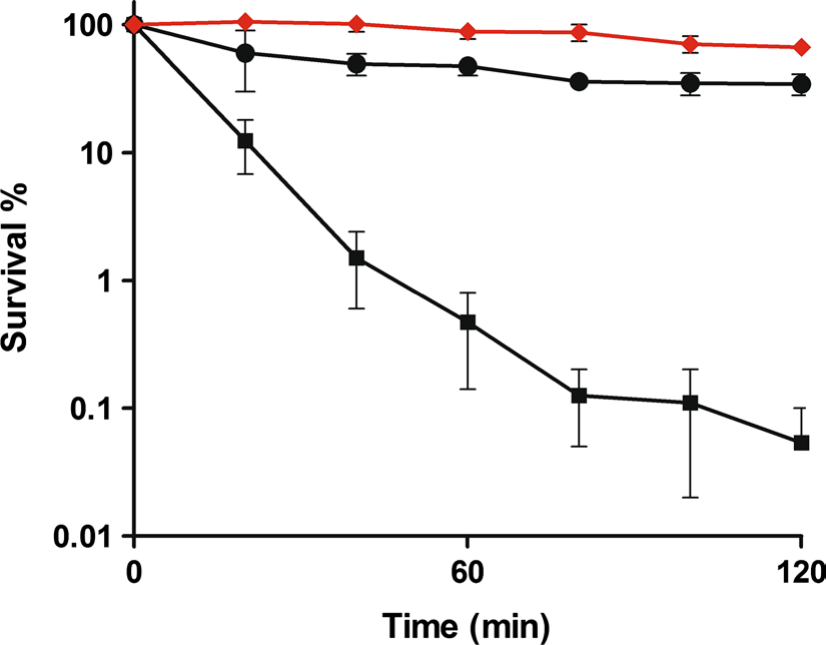
Induced C-6-H resistance of SH1000 by pre-incubation with a sub-MIC of C-6-H. SH1000 was grown in TSB^−Fe^ with or without 10 µg/ml C-6-H until OD_600_ 0.5–0.6. Cells were harvested, washed with dH_2_O and challenged with 0 (*filled circle*), 10 (*filled square*) or 10 with preincubation (*filled diamond*) µg/ml C-6-H over 2 h. Samples were taken, and cfu was determined over time. Samples were plated in triplicate, and each experiment was repeated twice. *Error bars* indicate the standard error of the mean

**Fig. 3:**
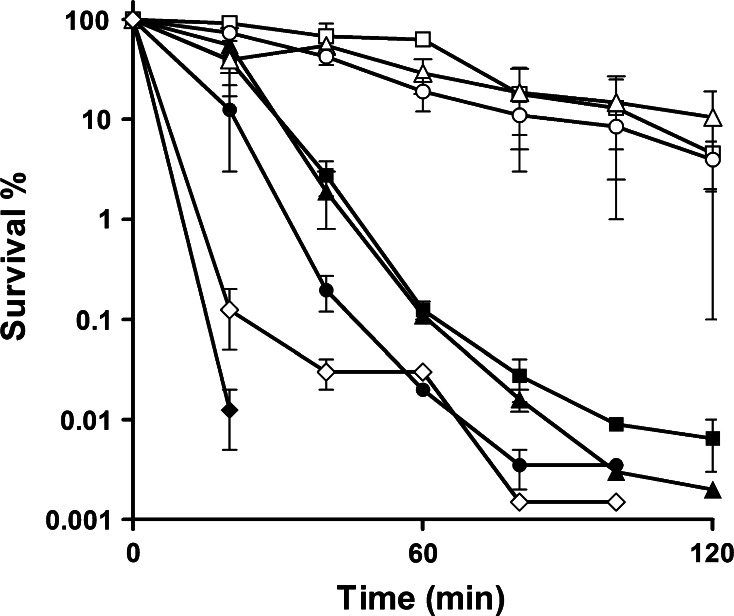
Bactericidal effect of C-6-H on cells preincubated with a sub-MIC concentration. SH1000 was grown in TSB^−Fe^ with (*open symbols*) or without (*filled symbols*) 8 µg/ml C-6-H until OD_600_ 0.5–0.6, as described in chapter 2. Cells were harvested, washed with dH_2_O and challenged with 30 (*open square*, *filled square*), 40 (*open triangle*, *filled triangle*), 50 (*open circle*, *filled circle*) or 60 (*open diamond*, *filled diamond*) µg/ml C-6-H. Samples were taken, and cfu was determined over time. Samples were plated in triplicate, and each experiment was repeated twice. *Error bars* indicate the standard error of the mean

To determine whether *S. aureus* responds to C-6-H, the effect of preincubation with a sub-MIC level of the fatty acid on resistance was determined. Firstly, to confirm the bactericidal effect of C-6-H, SH1000 was grown in TSB^−Fe^ to early exponential phase and challenged with 10 or 20 µg/ml C-6-H over 2 h. We observed a rapid decrease in survival rate. At *t* = 40 min, cells treated with 10 µg/ml showed only 1.3 % survival and cells treated with 20 µg/ml C-6-H showed a survival rate of 1 % (Fig. 1). The untreated control showed over 55 % survival after 120 min. Conversely, those cells previously exposed to subgrowth inhibitory level of C-6-H were much more resistant than naïve cells. After 1 h, 99 % of naïve cells were dead compared with only 12 % of those pretreated (Fig. 2). This indicates that *S. aureus* responds to C-6-H treatment by the induction of a resistance mechanism. In order to find out the level of C-6-H-induced resistance, additional assays were performed. C-6-H pretreated cells were incubated with 30, 40, 50 and 60 µg/ml C-6-H in the killing assay (Fig. 3). Pretreated cells showed resistance up to 50 µg/ml C-6-H challenge. Challenge with 50 µg/ml C-6-H killed over 99.9 % of naïve cells after 60 min of incubation, whereas pretreated cells were 80 % killed after this time. However, once 60 µg/ml C-6-H was used the induced resistance threshold was reached. There is no difference in sensitivity to C-6-H between the preincubated and non-preincubated cells (Fig. 3). Thus, a resistance mechanism is induced in response to C-6-H. *S. aureus* is an extremely adaptable organism able to respond to environmental assault. Chan and Foster (1998) reported an increase in resistance to acid stress (pH 2), when the cells were pre-exposed to non-lethal pH 4. How cells become more resistant to C-6-H is unknown, but no alteration in solvent partitioning by the cells was seen (data not shown). Cells grown in iron-limited conditions (as here) express *isdA*, which renders them hydrophilic (Clarke et al. 2007). IsdA is a major surface protein and has multiple roles as an adhesin, as a resistance determinant against human innate defences (including C-6-H), and is required for *S. aureus* survival on human skin (Clarke et al. 2004, 2007; Clarke and Foster 2008; Clarke 2010).

### Effect of C-6-H on global gene transcription

In order to determine mechanisms involved in response to C-6-H, including induction of resistance and inhibition of virulence determinant production, transcriptional profiling was carried out. The expression patterns from exponential phase culture of *S. aureus* SH1000 with and without 10 µg/ml C-6-H were compared. Under these conditions, C-6-H has no significant effect on growth rate or yield. Culture samples for analysis were taken 10 min and 60 min after C-6-H addition. After 10-min incubation with C-6-H, 290 genes were decreased and 293 genes were increased in expression level (Table S1). Besides many genes of unknown function and those encoding hypothetical proteins, there were also many genes whose expression was altered by C-6-H which encode ABC transporters, bacterial secretion systems, cell wall metabolism components, DNA replication and repair pathways as well as central metabolic and pathogenicity determinants. After 60-min incubation with C-6-H, 57 genes were reduced and 92 genes were increased in expression level compared with the untreated control (Table S1). These include genes encoding proteins involved in ABC transporter systems, metabolism of coenzymes and prosthetic groups, amino acid synthesis, stress response and many genes involved in virulence of *S. aureus.*

#### Effects of C-6-H on purine and pyrimidine biosynthesis

The greatest decrease in expression after 10-min C-6-H incubation was shown by genes encoding proteins involved in purine and pyrimidine biosynthesis (Table S1). In particular*, pyrR*, *pyrP, pyrB, pyrC, pyrAA, pyrAB*, *pyrF* and *pyrE* of the *de novo* pyrimidine metabolism were significantly down-regulated (spot vol. ratio 0.09 ± 0.04) in the presence of C-6-H compared with the control. This whole operon is regulated by *pyrR* and strongly depends on the presence of pyrimidine nucleotides (Turner et al. 1994; Paulus et al. 1982). Synthesis of pyrimidine is crucial for the cells to divide and therefore cell survival (Turnbough and Switzer 2008). The regulator *pyrR* showed a decrease in expression (spot vol. ratio of 0.06) in the presence of C-6-H (10-min incubation). Interestingly, after 60-min incubation with C-6-H, the operon *pyrR*-*E* (SA1041–SA1048) was highly increased in expression (Table S1). The regulator *pyrR* is increased in expression (spot vol. ratio 19.6), and genes *pyrP*, *pyrB, pyrC* and *pyrAB* were even more (spot vol. ratio 25 ± 5) up-regulated in expression, suggesting that the rapid response (10 min) of *S. aureus* to C-6-H may alter the intracellular pyrimidine pool, thus requiring increased expression of the genes after the initial adaptation period. Interestingly, the response to C-6-H may be specific as Kenny et al. (2009) reported no effect of linoleic or oleic acid on expression of genes involved in pyrimidine biosynthesis. How C-6-H and other fatty acids effect bacteria and differential responses alludes to fatty acid-specific mechanisms.

Genes that are involved in purine metabolism such as *xprT* (xanthine phosphoribosyltransferase), *guaA* (GMP synthase), *guaB* (inositol-monophosphate dehydrogenase) and *relA(rsh)* (GTP pyrophosphokinase) also showed a strong decrease in expression in the presence of C-6-H after 10-min incubation (spot vol. ratio 0.3 ± 0.2) (Table S1). After 60-min C-6-H incubation, the expression of *xprT, guaA, guaB* but not *relA* was still decreased. Interestingly, RelA as well as enzymes of the purine biosynthetic pathway has been described to be involved in resistance to lysostaphin (Gründling et al. 2006). Further, RelA (Rsh) may be important in the response to C-6-H as it effects the expression of a number of genes, including *cap* (via CodY) (Geiger et al. 2010; Pohl et al. 2009; Srivatsan and Wang 2008; Wolz et al. 2010).

### Effects of C-6-H on cellular transport systems

The microarray data demonstrated that many genes encoding for ABC transporters were effected in expression in the presence of C-6-H. Two genes, *cbiO* (Cobalt import ATP-binding protein Cibo1) and *cibO2* (Cobalt import ATP-binding protein CibO2), which are part of the cobalt transporter, are decreased in expression. After 10-min incubation with C-6-H, microarray data showed a spot vol. ratio of 0.42 and 0.49. Cobalt is an essential cofactor for several enzymes and other components such as vitamin B12 and must be imported into the cell (Kobayashi and Shimizu 1999). Surprisingly, other genes involved in cobalt transport such as *cbiQ* (transmembrane component) and *cbiN* (small membrane-bound component) were not affected in expression by C-6-H. After 60-min incubation with C-6-H, the *cbiO* genes showed no change in expression compared with the control (Table S1).

The expression of three genes, *potA*, *potB* and *potC*, from the *potABCD* operon were down-regulated after 10-min C-6-H incubation with a spot vol. ratio of 0.28 ± 0.05. After 60-min incubation with C-6-H, the expression of genes from the *potABCD* operon showed no change compared with the control. The operon encodes an ABC transporter that transports polyamines such as putrescine and spermidine across the membrane. Polyamines play an important role in cell proliferation and differentiation, as shown in *E. coli* (Kashiwagi et al. 1993).

Interestingly, the genes encoding for a putative monovalent cation/H^+^ antiporter were increased in expression in the presence of C-6-H (spot vol. ratio of 2.1 ± 0.1). SA0578 (putative antiporter subunit A), SA0579 (putative antiporter subunit B), SA0580 (putative antiporter subunit C), SA0581 (putative antiporter subunit D), SA0583 (putative antiporter subunit E) and SA0584 (putative antiporter subunit F) may be part of a Na^+^/H^+^ antiporter which is involved in resistance to high concentrations of K^+^, Li^+^ and Na^+^ ions. Antiporters play an important role in circulating Na^+^ and H^+^ across the cytoplasmic membrane (Padan and Schuldiner 1994) and are also important for the internal pH maintenance. Recently, we reported the bactericidal mechanisms of C-6-H at different concentrations (Cartron et al. 2014). C-6-H has multiple effects on the cell membrane including loss of the ability to maintain intracellular pH.

Compared with the genes that were affected after 10-min incubation, a completely different set was affected (positively as well as negatively) in expression after incubation with C-6-H for 60 min. The array data revealed that the genes *sirA*, *proP*, *fhuA*, *glpF* and SA2339 were increased in expression by C-6-H incubation. Interestingly, the gene SA2339 is highly up-regulated (spot vol. ratio over 11) in expression in the presence of C-6-H. SA2339 is, according to the database (NCBI), a hypothetical protein which shows similarities to an antibiotic transport-associated protein. The *sirA* gene (staphylococcal iron regulator) encodes for a lipoprotein, and the microarray data revealed a spot vol. ratio of 2.9, only after 60-min C-6-H incubation. The *sirA* gene is part of the operon *sirABC*. Heinrichs et al. (1999) reported that SirA acts as a membrane-associated siderophore-binding protein. The operon is iron regulated, and transcription is controlled by the Fur protein (Heinrichs et al. 1999). Interestingly, the regulatory gene *fur* showed a down-regulation (spot vol. ratio 0.48) after 10-min incubation with C-6-H. The *fur* gene encodes for the ferric uptake regulator which controls cellular iron homoeostasis (Horsburgh et al. 2001).

Furthermore, the gene *fhuA* (ferrichrome transport ATP-binding protein) was increased in expression after 60-min C-6-H incubation (spot vol. ratio 2.4). The *fhuA* gene is part of the *fhu* system which encodes for a ferric hydroxamate uptake system and therefore involved in iron acquisition from hydroxamate siderophore (Sebulsky et al. 2000). Interestingly, none of the other genes that belong to the *fhu* system were affected in expression by C-6-H. The *fhuD2* gene (spot vol. ratio 3.9) showed an increase in expression after 60-min C-6-H treatment. Sebulsky et al. (2003) reported that *fhuD2* encodes an iron (III)-siderophore-binding protein. After binding a siderophore, the FhuD2–siderophore complex will be recognised by the Fhu system. Iron is one of the most important nutrients for *S. aureus* and is required for various key metabolic processes. Its acquisition is vital for survival. The *sirABC* operon as well as the *fhu* genes is crucial components in the iron acquisition process.

The gene *proP*, which encodes for a proline/betaine transporter homologue (MacMillan et al. 1999), was increased (spot vol. ratio 2.4) in expression in the presence of C-6-H at 60 min only (Table S1). This transporter is important in balancing osmotic differences between *S. aureus* and its environment. The *proP* gene is part of the VraSR regulon, which includes 13 genes in total and is involved in regulation of the cell wall biosynthesis pathway (Kuroda et al. 2003).

### Effects of C-6-H on the cell envelope and cell wall synthesis

The expression of many genes involved in cell envelope biogenesis, including *lytM,* *dltABD*, *cidA*, *pbp2* and *pbp4*, was altered in response to C-6-H treatment. The peptidoglycan hydrolase LytM (autolysin) plays a role in cell wall turnover as well as cell division (Ramadurai et al. 1999). After 10-min incubation with C-6-H, the expression of *lytM* was decreased (spot vol. ratio 0.48) in expression, whereas after 60-min incubation no change in expression was observed. LytM hydrolyses peptidoglycan, and it has been proposed that it might play a role in the lysis of cells initiated by cell wall-acting antibiotics (Kusser and Ishiguro 1988), suggesting that C-6-H may induce lysis.

Genes of the *dltABCD* operon, which is important for d-alanylation of wall teichoic acids (WTA) as well as lipoteichoic acids (LTA), showed a strong decrease in expression in the presence of C-6-H after 10-min treatment. After 60-min C-6-H incubation, no change in expression of the *dltABCD* was observed. The *dltA* gene (spot vol. ratio 0.15) encodes a d-Alanyl carrier protein ligase and activates d-alanine using ATP. Also, *dltB*, a predicted transmembrane protein, and *dltD*, a membrane protein (Neuhaus and Baddiley 2003), showed a spot vol. ratio of 0.25 ± 0.02. Surprisingly, *dltC* expression showed no effect of C-6-H in the microarray data. Koprivnjak et al. (2006) reported that the *dltABCD* operon is highly repressed by a rising concentration of monovalent and divalent (Mg^2+^) cations. This group also mentioned that the transcriptional regulation may be partly due to the ArlSR two-component system. (Koprivnjak et al. 2006)

Weidenmaier et al. (2005) reported the role of the *dltABCD* operon in mediating resistance to cationic antimicrobial peptides (CAMP). DltABCD is able to modify negatively charged cell envelope components with positively charged amino acids, to enhance the net positive surface charge of *S. aureus* leading to CAMP resistance (Peschel 2002). The down-regulation (due to C-6-H) of *dltABD* expression may affect surface charge. Kohler et al. (2009) showed that wall teichoic acids, which play an important role in *S. aureus* surface charge, are crucial for protection of *S. aureus* against human skin fatty acids such as C-6-H. However, the treatment with C-6-H showed a strong decrease in expression of the *dltABCD* operon within the first few minutes but then recovers.

Penicillin-binding proteins 2 and 4 had altered expression (spot vol. ratios of 2.04 and 0.46, respectively) after 10-min incubation with C-6-H. PBP2 is involved in cell wall metabolism and methicillin resistance (Giesbrecht et al. 1998), and PBP4 is mainly involved in secondary cross-linking of the peptidoglycan layer (Henze and Berger-Bachi 1996), further suggesting that the cell wall of *S. aureus* is involved in protection against fatty acids.

The regulator *lytSR*, which encodes for a sensor histidine kinase (LytS) and a response regulator (LytR), was reduced (spot vol. ratio 0.38 ± 0.04) after 10-min C-6-H incubation (Table S1). LytSR is a sensor–regulator system with both positive and negative regulatory effects on murein hydrolase activity and autolysis (Brunskill and Bayles 1996). It is proposed that LytSR is able to sense a decrease in membrane potential and initiates the transcription of *lrgAB* (Patton et al. 2006; Bayles 2007). The *lrgAB* genes are involved in the regulation of murein hydrolase activity and may play a role in autolysin regulation (Groicher et al. 2000; Bayles 2003). Further studies reported that the *cidA* gene encodes for a holing-like membrane protein that is an effector of murein hydrolase activity and cell lysis, whereas *lrgA* encodes an antiholin that is an inhibitor of CidA (Groicher et al. 2000; Rice et al. 2003). Interestingly, the array data showed that the expression of *lrgAB* is highly increased after 10-min incubation with C-6-H (spot vol. ratios of 89 and 20, for *lrgA* and *lrgB*, respectively) (Table S1) even if its regulatory activator (*lytSR*) is inhibited in expression by C-6-H. LrgA and LrgB are involved in regulation of peptidoglycan hydrolase activity by reducing extracellular activity (Groicher et al. 2000; Bayles 2003). Bayles (2007) suggested that LrgAB together with their antagonist proteins CidAB was involved in the control of bacterial death and lysis during biofilm formation. Interestingly, the expression of *cidA* was decreased after 10-min C-6-H incubation (spot vol. ratio over 4). The *cidA* gene is in an operon with *cidB*. The *cidB* gene showed no change in expression levels in the presence of C-6-H, but *cidA* was decreased in expression and *cidC* was increased in its expression, respectively.

Further, Rice et al. (2003) showed that a *lytSR* mutation leads to an increase in autolysis rate and a decrease in *lrgAB* expression. These data were confirmed when Sharma-Kuinkel et al. (2009) reported that a mutation in the *lytS* gene showed a drastic decrease in the expression of *lrgAB* operon. Interestingly, in this study, the expression of *lytSR* showed a decrease in its transcription in the presence of C-6-H and the expression of the *lrgAB* operon was highly increased in its expression, suggesting that there might be an additional so far unknown regulatory system involved in *lrgAB* control.

We have observed that the bactericidal activity of C-6-H occurs via a variety of mechanisms, including loss of proton motive force (PMF) (Cartron et al. 2014), which may lead to an increase in *lytSR* expression.

The biological role of the *lrg* and *cid* operons has been suggested to be in the control of cell death and lysis during biofilm formation, as well as release of genomic DNA to promote intracellular adhesion in biofilm stability (Bayles 2007). The increased *lrgAB* expression and decreased expression of *cidA* may be due to cell wall stress and is a response that may prevent cell death due to metabolic perturbation. In a separate study, Kenny et al. (2009) observed a decrease of *lrgA* expression in response to linoleic acid, which demonstrates distinct responses to specific unsaturated long-chain fatty acids by *S. aureus*.

Capsule biosynthesis genes (*cap*) showed a significant increase in expression (spot vol. ratio of 2.4–7) (Table S1). The capsule is involved in the pathogenicity of *S. aureus* by preventing phagocytosis and killing by macrophages (O’Riordan and Lee 2004). Thus, it was possible that the capsule may represent a C-6-H resistance mechanism. Strain SH1000 is not highly encapsulated, and so the role of capsule was determined using strain Reynolds. Killing assays using strain Reynolds and corresponding *cap* strains did not show any significant differences in C-6-H susceptibility (data not shown). Kenny et al. (2009) also observed an increase in *cap* gene expression in response to linoleic acid by strain MRSA252. As the capsule has an antiphagocytic effect, it may be that host-associated stresses (such as C-6-H) lead to its induction as a general response.

### Effects of C-6-H on the expression of virulence determinants

Clarke et al. (2007) have previously reported a decrease in toxin production in response to C-6-H. The microarray data revealed altered expression of many genes involved in pathogenicity. After incubation with C-6-H, virulence-associated genes such as *nuc,* *hla, hlb*, *rsbU*, *sarZ, sarA, clfA* and two-component regulator *saeRS* were effected in expression (Table S1).

The thermonuclease (*nuc*) is an extracellular protein that degrades double- and single-stranded DNA and RNA. After 10-min incubation with C-6-H, the expression of *nuc* was decreased (spot vol. ratio 0.3), but after 60-min treatment with C-6-H the expression of *nuc* was increased (spot vol. ratio 2.4). The degradation of extracellular DNA plays an important role in host immune evasion to escape from neutrophils extracellular traps (NETs) (Berends et al. 2010).

Besides, the two-component regulator *saeRS* that encodes for a histidine kinase and a response regulator was affected by -6-H (Giraudo et al. 1999). After 10-min incubation with C-6-H, the expression of *saeRS* genes was decreased (spot vol. ratio 0.4). Novick and Jiang (2003) reported two additional ORFs in the SaeRS system, *saeP* and *saeQ*, which are likely to be important for the function of the operon (Adhikari and Novick 2008; Geiger et al. 2008). The *saeP* gene, but not *saeQ*, was decreased in expression (spot vol. ratio 0.28) after incubation with C-6-H for 10 min.

Previous reports have shown that SaeRS activates the expression of α-haemolysin (*hla*), β-haemolysin (*hlb*), fibronectin-binding protein (*fnbA* and *fnbB*), protein A (*spa*), coagulase (*coa*), thermonuclease (*nuc*), extracellular adherence protein Eap, IgG-binding protein (*sbi*) and extracellular matrix-binding protein Emp (Giraudo et al. 1994, 1997; Goerke et al. 2001, 2005; Harraghy et al. 2005). Furthermore, it represses the expression of V8 serine protease (*sspA*) and capsular polysaccharide (type 5) (*cap5*). As many of those virulence determinants (e.g. *hla, hlb, fnbA, nuc*) were decreased in expression due to C-6-H, this may be due to the activity of SaeRS (Table S1).

There was differential expression of surface proteins in response to C-6-H. Clumping factor A (*clfA*), a fibrinogen-binding protein, showed a high increase in expression after 10-min C-6-H incubation (spot vol. ratio 6.5). The ClfA protein plays an important role in the adhesion to host cells (McDevitt et al. 1994) and is responsible for clumping in host blood plasma (McDevitt et al. 1997). Kenny et al. (2009) reported a similar observation, where *clfA* expression is up-regulated. Interestingly, the surface protein-encoding genes *fnbA* and *fnbB* were decreased in expression by C-6-H. FnbA and B are cell surface-associated proteins which mediate the attachment to host cells (e.g. endothelial cells) and can also act during invasion (Peacock et al. 1999; Xu et al. 2008).

As already reported, the *hla* and *hlb* genes were highly decreased in expression after 60-min C-6-H incubation (spot vol. ratio 0.11 ± 0.02). These encode the major toxins α-haemolysin (*hla*) and β-haemolysin (*hlb*), which are membrane-damaging molecules, expressed in post-exponential phase, and are important for spreading into new host tissues (Bhakdi and Tranum-Jensen 1991). The effect of C-6-H on *hla* expression was expected as earlier reports have shown that fatty acids (e.g. glycerol monolaurate (GML) were able to inhibit the expression of virulence determinants in *S. aureus* (Schlievert et al. 1992; Projan et al. 1994). Clarke et al. (2007) found that C-6-H is able to inhibit the expression of α-haemolysin (*hla*) and protein A (*spa*) at the transcriptional level. Since no change in RNAIII expression (*agr*, the main regulator of virulence factors) has been observed after the exposure to C-6-H or GML, it leads to the suggestion that the effect of C-6-H on the expression of virulence determinants is due to another regulatory system (Projan et al. 1994; Clarke et al. 2007). In contrast, Kenny et al. (2009) observed that the expression of *hla* and *spa* was up-regulated after exposure of MRSA252 to linoleic or oleic acid.

The expression of *sbi* (IgG-binding protein) was negatively affected by C-6-H (spot vol. ratio 0.09 and 0.18 and after 10- and 60-min incubation, respectively). Sbi has an important role in adherence to host cells during the infective process and acts similarly to protein A (Zhang et al. 1998).

### Effects of C-6-H on general intracellular processes

Microarray data revealed altered expression of genes involved in carotenoid biosynthesis. The genes *crtN* (squalene desaturase), *crtM* (squalene synthase), *crtQ* (putative glycosyl transferase), *crtP* (4,4′-diaponeurosporene oxidase) and SA2352 (hypothetical protein) were increased in expression after incubation for 10 min with C-6-H (spot vol. ratio 3.7 ± 1.7). It has been reported that there is a direct correlation between carotenoid production and cell membrane fluidity (Chamberlain et al. 1991; Mishra et al. 2009). The carotenoids insert into the membrane and increase its rigidity, which could lead to its stabilization in response to C-6-H. Previous studies reported that long-chain unsaturated fatty acids could lead to an increase in membrane fluidity (Chamberlain et al. 1991). In the presence of the fatty acid C-6-H, the transcriptome data also showed an increase in expression of genes involved in fatty acid biosynthesis (*fabD, fabG, acpP*) after 60-min challenge conditions with C-6-H (spot vol. ratio 2.1 ± 0.1) (Table S1). The synthesis of fatty acids is essential for membrane phospholipid formation and stability, suggesting that *S. aureus* responds to the potential loss of membrane integrity due to C-6-H by increasing the expression of genes involved in fatty acid biosynthesis.

Several genes involved in stress responses were affected in expression by C-6-H incubation. The *katA* gene encodes catalase and is increased in expression only after 10-min incubation with C-6-H (spot vol. ratio 5). It is important for *S. aureus* for neutralisation of H_2_O_2_, survival and nasal colonisation (Cosgrove et al. 2007).

The microarray data revealed that genes of the Clp family (*clpC*, *clpB* and *clpL*) were increased in expression in the presence of C-6-H (spot vol. ratio 2.4, 3.7 and 13.1, respectively). ClpC is a chaperone with ATPase activity, and together with ClpB, it is highly induced during thermal stress with a function to degrade heat-damaged proteins, as well as an important role in biofilm formation (Becker et al. 2001; Frees et al. 2003). ClpB is required for growth at high temperature (Frees et al. 2003). ClpL is also an ATP-dependent proteinase and is important in the thermotolerance of *S. aureus* (Frees et al. 2003).

In response to C-6-H, there is an up-regulation of urease (*ureA*-*G*) expression (60-min incubation, spot vol. ratio 4 ± 2, Table S1). Urease is involved in hydrolysing urea into NH_3_ (ammonia) and CO_2_ (carbon dioxide) as a nitrogen source and acid resistance.

The transcription of *dhoM* (homoserine dehydrogenase), *thrC* (threonine synthetase) and *thrB* (homoserine kinase) was increased after incubation with C-6-H for 10 min (spot vol. ratio 7.5 ± 3.5) and for 60 min (spot vol. ratio 3.1 ± 0.2). All three proteins are important for amino acid synthesis such as serine, glycine or threonine. ThrC catalyses the last reaction of threonine synthesis from asparate.

### Real-time PCR (RT-PCR)

The transcriptome data of selected genes were validated using RT-PCR (Table 3), whereas all samples were amplified in triplicate. The focus was on genes involved in virulence and stress response. The RT-PCR analysis showed that *hla* was 20-fold down-regulated after 60-min incubation with C-6-H. For the genes *lrgA, lrgB, katA, sarA*, *sceD, cidA, lytS* and *butA*, RT-PCR data also confirmed the microarray results, verifying the high impact of C-6-H on the expression of those genes. However, not all qRT-PCR data are in correlation with the effect of C-6-H observed in the microarray data. For *spa*, *hprT*, *phoP*, *asp23*, *lytR* and *saeRS*, no effect on expression due to C-6-H was observed (Table 3). This demonstrates that multiple approaches should be undertaken to verify changes in expression using these types of technologies.

**Table 3:** Effect of C-6-H on expression of genes determined by qRT-PCR

ORF N315	Gene	Gene product	Fold change RT-PCR		Spot vol ratio (microarray)	
			10 min	60 min	10 min	60 min
Cell envelope and cell wall						
SA0252	*IrgA*	Holin-like protein LrgA	5.00	0.27	89.97	1.00
SA0253	*IrgB*	Holin-like protein LrgB	4.00	0.43	20.73	1.00
SA0265	*IytM*	Peptidoglvcan hydrolase	1.00	3.00	0.49	1.00
SA2329	*cidA*	Holin-like protein CidA	0.23	1.00	0.22	1.00
Virulence factors and regulators						
SA0250	*lytS*	Two-component sensor histidine kinase	0.44	1.00	0.43	1.00
SA0251	*lytR*	Two-component response regulator	1.00	1.00	0.35	1.00
SA0573	*sarA*	Staphylococcal accessory regulator A	2.00	1.00	2.37	1.00
SA0660	*saeS*	Histidine protein kinase	1.00	1.00	0.44	1.00
SA0661	*saeR*	Response regulator	1.00	1.00	0.41	1.00
SA0742	*clfA*	Fibrinogen-binding protein A, clumping factor	1.00	1.00	6.54	1.00
SA1007	*hla*	Alpha-haemolysin	0.41	0.07	1.00	0.13
SA1842	*agrB*	Accessory gene regulator B	3.00	1.00	1.00	1.00
SA1872	*rsbU*	SigmaB regulation protein RsbU	1.00	1.00	0.49	1.00
Stress response						
SA1170	*katA*	Catalase	3.00	1.00	5.06	1.00
SA1984	*asp23*	Alkaline shock protein 23, ASP23	1.00	1.00	7.52	1.00
Butanoate metabolism						
SAO122	*butA*	Acetoin reductase	5.00	1.00	16.89	2.72
Transcriptional regulator						
SA0641	*mrzA*	HTH-type transcriptional regulator MgrA (NorA)	1.00	1.00	0.50	1.00
SA1515	*phoR*	Alkaline phosphatase synthesis sensor protein	1.00	1.00	1.00	1.00
SA1516	*phoP*	Alkaline phosphatase synthesis transcriptional regulation	1.00	1.00	3.31	1.00
Purine and/ or pyrimidine metabolism						
SA0468	*hprT*	Hypoxanthine-guanine phosphoribosyltransferase homologue	1.00	1.00	0.29	1.00
Unknown functions and hypothetical proteins						
SA1898	*sceD*	Hypothetical protein, similar to SceD precursor	1.00	3.00	1.00	3.18
Housekeeping genes						
SA0005	*gyrB*	DNA gyrase subunit B	1.00	1.00	1.00	1.00
SA1186		Hypothetical protein, homologue toyneS from B. subtilis	1.00	1.00	1.00	1.00
SA1497	*ysxC*	Ribosome biogenesis GTP-binding protein YsxC	1.00	1.00	1.00	1.00

### Effect of C-6-H on the *S. aureus* regulatory system *saeRS*

**Fig. 4:**
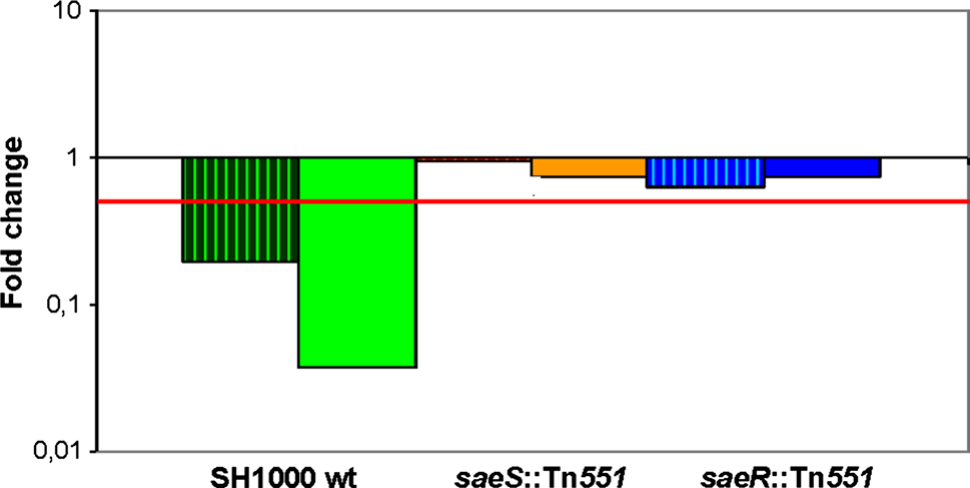
Effect of C-6-H on *hla* expression in *saeRS* mutant strains. *S. aureus* SH1000 wt, SH1000 *saeS*∷Tn*551* and SH1000 *saeR*∷Tn*551* were grown in TSB^−Fe^ until OD_600_ 0.5. 8 µg/ml C-6-H was added to the cultures, and cells were incubated for 10 (*striped bars*) or 60 (*filled bars*) min. Total mRNA was isolated, and an qRT-PCR experiment was performed determining *hla* expression. *Red line* shows significant change of at least 0.5-fold. The samples were measured in triplicate, and qRT-PCR experiment was carried out three times

The SaeRS response regulator is a key system that controls the expression of virulence determinants in *S. aureus* and is required for pathogenesis (Giraudo et al. 1994, 1997; Goerke et al. 2001, 2005; Harraghy et al. 2005). Interestingly, *saeRS* was down-regulated in response to C-6-H as shown by the transcriptome data. Further, many members of its regulon were decreased in expression as well, which suggests that signal transduction by SaeS may be altered as a direct, or indirect, consequence of C-6-H. It has been proposed earlier that fatty acids (such as GML or lauric acid) might affect the cell membrane and therefore disrupt important signalling mechanisms (Schlievert et al. 1992; Holland et al. 1994; Projan et al. 1994; Ruzin and Novick 1998; Vetter and Schlievert 2005). To investigate the role of the SaeRS system in the molecular mechanism of C-6-H, qRT-PCR experiments were performed. The transcription of *hla* in the presence of a sub-MIC of C-6-H using the *saeS* (*saeS*∷Tn*551*) and *saeR* (*saeR*∷Tn*551*) mutant strains in the SH1000 background was measured. In SH1000 wt, the expression of *hla* showed a reduction of over fourfold and 20-fold in the presence of C-6-H for 10 and 60 min, respectively. The *saeS* mutant strain (*saeS*∷Tn*551)* revealed no significant change in *hla* expression in the presence of C-6-H, at 10 or 60 min (Fig. 4). Similar results were observed using the *saeR* mutant strain (*saeR*∷Tn*551)* which also showed no significant difference in *hla* expression in the presence of C-6-H, suggesting that the two-component system *saeRS* is implicated in the molecular mechanism of C-6-H. The molecular mechanism of C-6-H in virulence determinant inhibition has not as yet been revealed, but an interference of the signal transduction system in *S. aureus* is a possible scenario.

### Effect of C-6-H on protein profiles

Transcriptome analysis revealed a pleiotropic effect of C-6-H on gene expression but whether this is translated into significant changes in protein levels and thus activities required a proteomic analysis. The effect of a sub-inhibitory concentration of C-6-H on the extracellular proteins of *S. aureus* SH1000 was determined using the 2D gel technique (Table 4).

**Table 4:** Growth phase associated changes in extracellular protein profile

ORF N315	Protein	Gene product	OD 1.0 versus 16 h	Spot ID
SA0009	SerS	Seryl-tRNA synthetase	0.65	331
SA0091	Plc	1-Phosphatidylinositol phosphodiesterase precurosr	2.29	563
SA0091	Plc	1-Phosphatidylinositol phosphodiesterase precurosr	4.83	565
SA0091	Plc	1-Phosphatidylinositol phosphodiesterase precurosr	38.19	571
SA0128	SodM (SodA1)	Superoxide dismutase	1.60	698
SA0131	Pnp (DeoD1)	Purine nucleoside phosphorylase	1.11	654
SA0131	Pnp (DeoD1)	Purine nucleoside phosphorylase	0.06	187
SA0162	AldA	Aldehyde dehydrogenase homologue	0.27	294
SA0182		Hypothetical protein, similar to indole-3-pyruvate decarboxylas	1.12	280
SA0265	LytM	Peptidoglycan hydrolase	0.15	454
SA0265	LytM	Peptidoglycan hydrolase	0.13	460
SA0309	Geh	Glycerol ester hydrolase	2.29	170
*SA0309*	*Geh*	*Glycerol ester hydrolase*	*3.08*	*171*
SA0309	Geh	Glycerol ester hydrolase	2.84	172
SA0309	Geh	Glycerol ester hydrolase	1.81	175
SA0309	Geh	Glycerol ester hydrolase	4.03	177
*SA0309*	*Geh*	*Glycerol ester hydrolase*	*9.64*	*199*
SA0309	Geh	Glycerol ester hydrolase	3.28	212
SA0309	Geh	Glycerol ester hydrolase	1.76	224
*SA0309*	*Geh*	*Glycerol ester hydrolase*	*21.07*	*229*
SA0309	Geh	Glycerol ester hydrolase	5.23	272
SA0309	Geh	Glycerol ester hydrolase	1.81	282
SA0309	Geh	Glycerol ester hydrolase	0.83	421
SA0309	Geh	Glycerol ester hydrolase	2.75	433
SA0309	Geh	Glycerol ester hydrolase	0.57	439
SA0309	Geh	Glycerol ester hydrolase	3.00	443
SA0309	Geh	Glycerol ester hydrolase	1.20	451
SA0309	Geh	Glycerol ester hydrolase	1.90	176
SA0309	Geh	Glycerol ester hydrolase	1.17	217
SA0309	Geh	Glycerol ester hydrolase	3.97	221
SA0309	Geh	Glycerol ester hydrolase	0.68	238
SA0309	Geh	Glycerol ester hydrolase	2.99	248
SA0309	Geh	Glycerol ester hydrolase	1.32	259
SA0309	Geh	Glycerol ester hydrolase	1.97	293
SA0309	Geh	Glycerol ester hydrolase	3.40	418
*SA0309*	*Geh*	*Glycerol ester hydrolase*	*8.89*	*424*
SA0309	Geh	Glycerol ester hydrolase	5.58	435
SA0309	Geh	Glycerol ester hydrolase	1.15	436
*SA0366*	*AhpC*	*Alkyl hydroperoxide reductase subunit C*	*3.04*	*712*
SA0375	GuaB	Inositol-monophosphate dehydrogenase	0.34	308
SA0376	GuaA	GMP synthase	2.00	274
SA0382	Set6	Superantigen-like protein	0.03	706
SA0482		Putative ATP: guanido phosphotransferase SA0482	0.76	366
SA0486	GltX	Glutamyl-tRNA synthetase	1.21	289
SA0486	GltX	Glutamyl-tRNA synthetase	0.41	302
SA0488	CysS	Cysteinyl-tRNA synthetase	0.64	309
*SA0505*	*FusA*	*Elongation factor G*	*0.14*	*162*
SA0506	Tuf	Elongation factor Tu	0.69	371
SA0506	Tuf	Elongation factor Tu	0.63	396
*SA0544*		*Putative haem peroxidase*	*0.21*	*618*
SA0587		Lipoprotein, streptococcal adhesin PsaA homologue	1.63	561
SA0587		Lipoprotein, streptococcal adhesin PsaA homologue	0.22	610
SA0620		Secretory antigen SsaA homologue	0.28	626
SA0674		Glycerol phosphate lipoteichoic acid synthase	0.87	336
SA0674		Glycerol phosphate lipoteichoic acid synthase	0.53	343
SA0674		Glycerol phosphate lipoteichoic acid synthase	1.15	344
SA0674		Glycerol phosphate lipoteichoic acid synthase	0.70	346
SA0674		Glycerol phosphate lipoteichoic acid synthase	0.50	353
SA0686	NrdE	Ribonucleotide-diphosphate reductase subunit alpha	0.51	189
SA0719	TrxB	Thioredoxin reductase	1.82	508
SA0727	Gap	Glyceraldehyde-3-phosphate dehydrogenase	0.54	447
SA0728	Pgk	Phosphoglycerate kinase	0.57	409
SA0731	Eno	Phosphopyruvate hydratase	0.55	384
SA0732	ClpP	ClpP	1.07	729
SA0775		Hypothetical protein	0.52	296
SA0787		IS1181 transposase	0.37	242
SA0802		NADH dehydrogenase-like protein SA0802	0.94	411
SA0820	GlpQ	Glycerophosphoryl diester phosphodiesterase	2.31	569
SA0820	GlpQ	Glycerophosphoryl diester phosphodiesterase	1.72	570
SA0823	Pgi	Glucose-6-phosphate isomerase	1.10	378
SA0829		Hypothetical protein	0.16	573
SA0831	Cdr	Coenzyme A disulphide reductase	2.08	349
SA0842	FabH	FabH, 3-oxoacyl-(acyl carrier protein) synthase homologue	1.04	489
SA0843	Fab (FabF)	3-oxoacyl-synthase	0.94	362
SA0900	SspB1	Cysteine protease precursor SspB	1.72	427
SA0900	SspB1	Cysteine protease precursor SspB	1.25	432
SA0900	SspB1	Cysteine protease precursor SspB	2.53	468
SA0900	SspB1	Cysteine protease precursor SspB	1.51	825
SA0901	SspA	V8 protease	0.77	474
SA0901	SspA	V8 protease	1.26	478
SA0901	SspA	V8 protease	1.30	483
SA0901	SspA	V8 protease	1.73	486
SA0901	SspA	V8 protease	0.39	507
SA0901	SspA	V8 protease	1.47	511
SA0901	SspA	V8 protease	1.37	490
SA0904	Atl	ATL autolysin transcription regulator	0.28	163
SA0908		Hypothetical protein	1.80	417
SA0908		Hypothetical protein	1.90	419
*SA0935*	*PtsI*	*Phosphoenolpyruvate-protein phosphatase*	*0.09*	*244*
*SA0939*		*Hypothetical protein*	*0.12*	*676*
*SA0945*	*PdhC*	*Branched-chain alpha-keto acid dehydrogenase subunit E2*	*0.47*	*192*
SA0946	PdhD	Dihydrolipoamide dehydrogenase	1.04	287
*SA1007*	*Hla*	*Alpha-haemolysin*	*5.28*	*531*
SA1007	Hla	Alpha-haemolysin	2.71	536
SA1007	Hla	Alpha-haemolysin	5.04	539
*SA1007*	*Hla*	*Alpha-haemolysin*	*4.60*	*541*
SA1007	Hla	Alpha-haemolysin	1.92	651
SA1036	IleS	Isoleucyl-tRNA synthetase	0.41	132
SA1098	CodY	Transcriptional repressor CodY	2.87	617
SA1099	RpsB	30S ribosomal protein S2	0.55	514
SA1100	Tsf	Elongation factor Ts	2.23	459
SA1100	Tsf	Elongation factor Ts	1.58	470
SA1128	RecA	Recombinase A	0.79	389
SA1150	GlnA	Glutamine–ammonia ligase	1.70	323
SA1170	KatA	Catalase	1.87	263
*SA1177*	*Tkt*	*Transketolase*	*0.40*	*197*
SA1177	Tkt	Transketolase	1.31	201
SA1177	Tkt	Transketolase	3.11	401
SA1533	AckA	Acetate kinase homologue	0.40	393
*SA1184*	*CitB (AcnA)*	*Aconitate hydratase*	*0.23*	*128*
SA1216	PepF	Hypothetical protein, similar to oligoendopeptidase	33.50	215
SA1283	Pbp2	PBP2	0.57	220
SA1308	RpsA	30S ribosomal protein S1	0.43	363
SA1336		Glucose-6-phosphate 1-dehydrogenase	1.50	250
SA1342	Gnd	6-Phosphogluconate dehydrogenase	2.22	391
SA1342	Gnd	6-Phosphogluconate dehydrogenase	2.20	400
SA1359	Efp	Elongation factor P	0.40	560
SA1409	DnaK	Molecular chaperone DnaK	0.69	226
SA1409	DnaK	Molecular chaperone DnaK	2.15	546
*SA1499*	*Tig*	*Trigger factor*	*0.10*	*231*
SA1520	PykA	Pyruvate kinase	0.68	203
SA1529		Metal-dependent hydrolase	6.88	669
SA1553	Fhs	Formate-tetrahydrofolate ligase	2.57	273
SA1553	Fhs	Formate-tetrahydrofolate ligase	1.16	277
SA1579	LeuS	Leucyl-tRNA synthetase	2.68	143
SA1599	Tal	Hypothetical protein, similar to transaldolase	1.17	659
SA1609	PckA	Phosphoenolpyruvate carboxykinase	2.05	279
SA1627	SplF	Serine protease SplE, putative	2.93	667
SA1627	SplF	Serine protease SplE, putative	7.46	660
*SA1627*	*SplF*	*Serine protease SplE, putative*	*7.88*	*670*
*SA1628*	*SplD*	*Serine protease SplD*	*4.68*	*666*
SA1629	SplC	Serine protease SplC	4.43	656
SA1629	SplC	Serine protease SplC	1.41	657
*SA1630*	*SplB*	*Serine protease SplB*	*6.64*	*646*
SA1631	SplA	Serine protease SplA	4.55	642
SA1631	SplA	Serine protease SplA	2.01	647
SA1637	LukD	Leukotoxin, LukD	1.22	487
SA1653	TRAP	Signal transduction protein TRAP	6.36	914
SA1695	AmpS	Aminopeptidase ampS	1.34	397
SA1709		Ferritin	0.32	910
SA1725	SspB2	Staphopain, cysteine proteinase	1.76	725
*SA1725*	*SspB2*	*Staphopain, cysteine proteinase*	*6.01*	*754*
SA1811	Hlb	Beta-haemolsysin	1.28	505
SA1811	Hlb	Beta-haemolsysin	1.02	509
SA1811	Hlb	Beta-haemolsysin	0.38	515
SA1811	Hlb	Beta-haemolsysin	5.31	519
SA1811	Hlb	Beta-haemolsysin	2.31	520
SA1811	Hlb	Beta-haemolsysin	0.29	522
SA1811	Hlb	Beta-haemolsysin	0.65	574
SA1812		Uncharacterized leukocidin-like protein 1 precursor	1.64	499
SA1812		Uncharacterized leukocidin-like protein 1 precursor	2.19	500
SA1812		Uncharacterized leukocidin-like protein 1 precursor	1.21	502
SA1813		Uncharacterized leukocidin-like protein 2 precursor	0.72	494
SA182	SodA (SodA2)	Superoxide dismutase SodA	1.53	697
SA1836	GroEL	Chaperonin GroEL	0.37	267
*SA1898*		*Hypothetical protein, similar to SceD precursor*	*0.27*	*552*
SA1905	AtpD	F0F1 ATP synthase subunit beta	0.28	383
SA1915	GlyA	Serine hydroxymethyltransferase	1.24	364
SA1915	GlyA	Serine hydroxymethyltransferase	0.90	367
SA1927	FbaA	Fructose-bisphosphate aldolase	0.61	530
SA1959	GlmS	Glucosamine-fructose-6-phosphate transferase	1.12	218
SA1984	Asp23	Alkaline shock protein 23	1.66	827
SA2003	HysA	Hyaluronate lyase precursor	0.30	156
*SA2093*	*SsaA*	*Secretory antigen precursor SsaA homolog*ue	*0.09*	*592*
*SA2093*	*SsaA*	*Secretory antigen precursor SsaA homologue*	*0.10*	*593*
SA2097		Hypothetical protein, similar to secretory antigen precursor SsaA	0.24	860
*SA2204*	*GpmA*	*Phosphoglycerate mutase, pgm homologue*	*3.01*	*583*
SA2204	GpmA	Phosphoglycerate mutase, pgm homolog*ue*	1.36	585
SA2206	Sbi	IgG-binding protein SBI	0.29	387
*SA2208*	*HlgC*	*Gamma-haemolysin component C*	*3.73*	*535*
*SA2209*	*HlgB*	*Gamma-haemolysin component B*	*2.47*	*497*
SA2334	MmvaS	3-Hydroxy-3-methylglutaryl CoA synthase	0.68	434
SA2336	ClpL	ATP-dependent Clp proteinase chain clpL	0.35	210
SA2356	IsaA	Immunodominant antigen A	0.23	616
*SA2356*	*IsaA*	*Immunodominant antigen A*	*0.22*	*635*
SA2356	IsaA	Immunodominant antigen A	2.08	747
SA2356	IsaA	Immunodominant antigen A	1.43	822
SA2356	IsaA	Immunodominant antigen A	0.24	908
SA2430	Aur	Zinc metalloproteinase aureolysin	0.21	471
SA2430	Aur	Zinc metalloproteinase aureolysin	0.79	496
SA2437		Hypothetical protein, similar to autolysin precursor	0.36	191
SA2437		Hypothetical protein, similar to autolysin precursor	0.19	193
*SA2437*		*Hypothetical protein, similar to autolysin precursor*	*0.16*	*195*
*SA2437*		*Hypothetical protein, similar to autolysin precursor*	*0.11*	*200*
SA2437		Hypothetical protein, similar to autolysin precursor	0.88	223
SA2437		Hypothetical protein, similar to autolysin precursor	1.11	235
SA2437		Hypothetical protein, similar to autolysin precursor	1.15	236
SA2437		Hypothetical protein, similar to autolysin precursor	0.10	245
SA2437		Hypothetical protein, similar to autolysin precursor	0.78	269
*SA2437*		*Hypothetical protein, similar to autolysin precursor*	*0.41*	*422*

Table of all identified protein spots from the extracellular fraction. Data for proteins with a spot vol. ratio of ≥2 and ≤0.5 are shown. All proteins had a significance level of 0.05 or less (*T* test 5 % cut-off). Proteins highlighted in italics are significantly changed in the two phases of growth

**Fig. 5:**
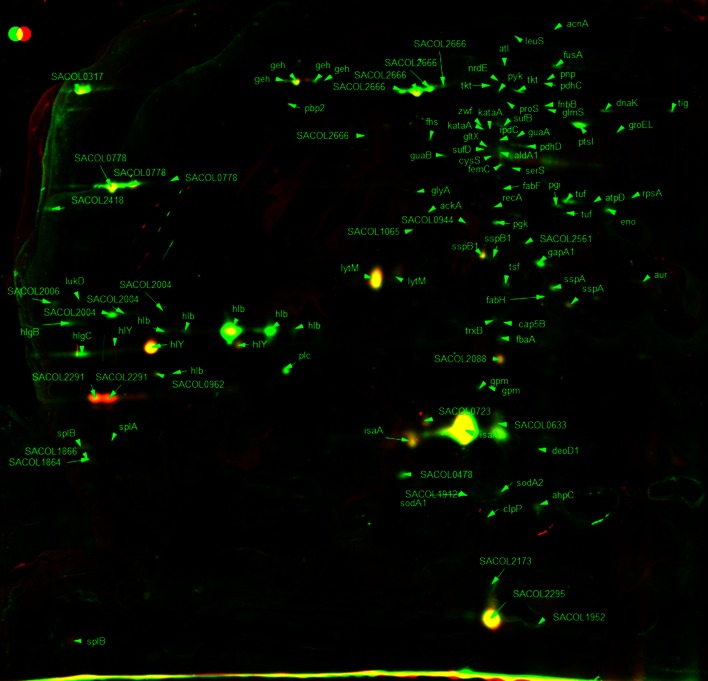
2D gel image false-colour dual-channel of extracellular proteins in exponential phase with and without C-6-H. Merged 2D gel images of *S. aureus* SH1000 extracellular proteins from exponential phase treated with or without 10 µg/ml C-6-H. Control gel shown in *green*, treated samples shown in *red* and equal expression shown in *yellow*. *Spots* were identified via MALDI-TOF

**Fig. 6:**
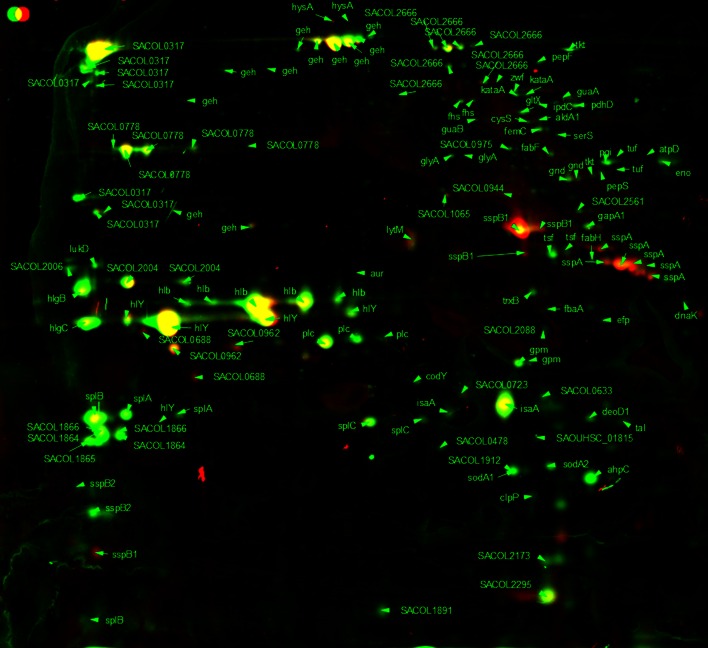
2D gel image false-colour dual-channel of extracellular proteins in stationary phase with and without C-6-H. Merged 2D gel images of *S. aureus* SH1000 extracellular proteins from stationary phase treated with or without 10 µg/ml C-6-H. Control gel shown in *green*, treated samples shown in *red* and equal expression shown in *yellow*. Spots were identified via MALDI-TOF

For the 2D gel analysis of extracellular proteins, culture supernatant was treated with 10 % (w/v) fresh TCA to precipitate all extracellular proteins. Figures 5 and 6 show the extracellular protein expression of *S. aureus* in early exponential phase (OD_600_ 1.0) and stationary phase (16 h incubation). In total, 103 different proteins were identified in the extracellular protein fraction. Nine proteins showed an increase in the amount in stationary phase and 13 proteins showed a decrease in level compared with exponential phase (Table 5). As expected, virulence determinants that are involved in host defence evasion as well as invasion and tissue penetration showed an increased level in stationary phase. For example, α-haemolysin (Hla) was over 4.5-fold increased in level in stationary phase. Also, the glycerol ester hydrolase (Geh) main spots were increased ninefold to 21-fold (Table 4). Decreased protein levels in stationary phase were shown by several hypothetical proteins as well as the peptidoglycan hydrolase (LytM), immunodominant antigen A (IsaA) and secretory antigen (SsaA).

**Table 5:** Growth phase-dependent changes in extracellular protein profile

ORF N315	Protein	Gene product	OD 1.0 versus 16 h	Spot ID
SA0265	LytM	Peptidoglycan hydrolase	0.15	454
SA0265	LytM	Peptidoglycan hydrolase	0.13	460
SA0309	Geh	Glycerol ester hydrolase	3.08	171
SA0309	Geh	Glycerol ester hydrolase	9.64	199
SA0309	Geh	Glycerol ester hydrolase	21.07	229
SA0309	Geh	Glycerol ester hydrolase	8.89	424
SA0375	GuaB	Inositol-monophosphate dehydrogenase	0.34	308
SA0393	Set15	Superantigen-like protein	0.12	676
SA0505	FusA	Elongation factor G	0.14	162
SA0544		Putative haem peroxidase	0.21	618
SA0935	PtsI	Phosphoenolpyruvate-protein phosphatase	0.09	244
SA0945	PdhC	Branched-chain alpha-keto acid dehydrogenase subunit E2	0.47	192
SA1007	Hla	Alpha-haemolysin	5.28	531
SA1007	Hla	Alpha-haemolysin	4.60	541
SA1177	Tkt	Transketolase	0.40	197
SA1499	Tig	Trigger factor	0.10	231
SA1627	SplF	Serine protease SplE, putative	7.88	670
SA1628	SplD	Serine protease SplD	4.68	666
SA1630	SplB	Serine protease SplB	6.64	646
SA1725		Staphopain, cysteine proteinase	6.01	754
SA1898		Hypothetical protein, similar to SceD precursor	0.27	552
SA2093	SsaA	Secretory antigen precursor SsaA homologue	0.09	592
SA2093	SsaA	Secretory antigen precursor SsaA homologue	0.10	593
SA2204	GpmA	Phosphoglycerate mutase, pgm homologue	3.01	583
SA2208	HlgC	Gamma-haemolysin component C	3.73	535
SA2209	HlgB	Gamma-haemolysin component B	2.47	497
SA2356	IsaA	Immunodominant antigen A	0.22	635
SA2437		Hypothetical protein, similar to autolysin precursor	0.16	195
SA2437		Hypothetical protein, similar to autolysin precursor	0.41	422

Comparison of the pattern of extracellular protein expression in exponential phase (OD_600_ 1.0) and stationary phase of *S. aureus*. Data for proteins with a spot vol. ratio of ≥2 and ≤0.5 are shown. All genes had a significance level of 0.05 or less (*T* test 5 % cut-off)

In early exponential phase (OD_600_ 1.0), the levels of 15 proteins were altered (≥twofold) in the presence of C-6-H (Table 6). Thirteen proteins showed a reduced level and 2 proteins showed an increased level in the presence of C-6-H. In stationary phase (16 h), 18 proteins showed an altered level of expression in the presence of C-6-H. Thirteen proteins were reduced and 5 were increased in level (Table 6). In the presence of C-6-H, several virulence determinants were reduced in level in exponential phase. The β-haemolysin (Hlb) and γ-haemolysin (HlgC) showed fivefold and 2.5-fold reduction in exponential phase. At stationary phase, Hlb showed no alteration due to C-6-H, but HlgC and HlgB were 3.5-fold reduced. The addition of C-6-H surprisingly showed no apparent effect on Hla level at either growth phase. The lipase (Geh) showed a growth phase-dependent response to C-6-H as it was reduced in exponential phase but induced in stationary phase (Table 5).

**Table 6:** Effect of C-6-H on extracellular protein profile

ORF N315	Protein	Gene product	Expression C6H			
			OD 1.0	16 h	Spot ID (OD 1.0)	Spot ID (16 h)
SA0131	Pnp (deoD1)	Purine nucleoside phosphorylase	–	0.33		654
SA0265	LytM	Peptidoglycan hydrolase	2.08	–	454	
SA0309	Geh	Glycerol ester hydrolase	0.23	–	421	
SA0309	Geh	Glycerol ester hydrolase	–	7.38		212
SA0366	AhpC	Alkyl hydroperoxide reductase subunit C	–	0.18		712
SA0505	FusA	Elongation factor G	0.20	–	162	
SA0506	Tuf	Elongation factor Tu	0.17	–	371	
SA0820	GlpQ	Glycerophosphoryl diester phosphodiesterase	–	2.88		569
SA0843	Fab (fabF)	3-Oxoacyl-synthase	0.10	–	362	
SA0900	SspB1	Cysteine protease precursor SspB	–	7.57		427
SA0900	SspB1	Cysteine protease precursor SspB	–	5.78		432
SA0901	SspA	V8 protease	–	13.24		478
SA0901	SspA	V8 protease	–	9.44		483
SA0901	SspA	V8 protease	–	5.19		490
SA0935	PtsI	Phosphoenolpyruvate-protein phosphatase	0.18	–	244	
SA1100	Tsf	Elongation factor Ts	0.47	–	470	
SA1177	Tkt	Transketolase	0.25	–	201	
SA1184	CitB (acnA)	Aconitate hydratase	0.13	–	128	
SA1409	Dnak	Molecular chaperone DnaK	0.19	–	226	
SA1627	SplF	Serine protease SplE, putative	–	0.36		670
SA1630	SplB	Serine protease SplB	–	0.29		646
SA1631	SplA	Serine protease SplA	–	0.27		642
SA1631	SplA	Serine protease SplA	–	0.13		647
SA1637	LukD	Leukotoxin, LukD	–	0.17		487
SA1671		Hypothetical protein	–	0.17		698
SA1725	SspB2	Staphopain, cysteine proteinase	–	0.19		754
SA1811	Hlb	Beta-hemolsysin	0.17	–	505	
SA1811	Hlb	Beta-hemolsysin	0.19	–	509	
SA1811	Hlb	Beta-hemolsysin	0.16	–	519	
SA1812		Hypothetical protein	–	0.44		500
SA1813		Hypothetical protein	–	0.07		494
SA1959	GlmS	Glucosamine-fructose-6-phosphate transferase	0.05	–	218	
SA2093	SsaA	Secretory antigen precursor SsaA homologue	4.74	–	592	
SA2093	SsaA	Secretory antigen precursor SsaA homologue	6.24	–	593	
SA2204	GpmA	Phosphoglycerate mutase, pgm homologue	0.08	–	585	
SA2208	HlgC	Gamma-haemolysin component C	0.38	–	535	
SA2208	HlgC	Gamma-haemolysin component C	–	0.33		535
SA2209	HlgB	Gamma-haemolysin component B	–	0.22		497
SA2356	IsaA	Immunodominant antigen A	–	0.28		616
SA2356	IsaA	Immunodominant antigen A	–	0.21		635
SA2356	IsaA	Immunodominant antigen A	–	0.16		747
SA2437		Hypothetical protein, similar to autolysin	–	0.43		223
SA2437		Hypothetical protein, similar to autolysin	–	2.38		236

Comparison of extracellular protein production in exponential phase (OD_600_ 1.0) and stationary phase in the presence of sub-MIC C-6-H. Data for proteins with a spot vol. ratio of ≥2 and ≤0.5 are shown. All proteins had a significance level of 0.05 or less (*T* test 5 % cut-off)

**Fig. 7:**
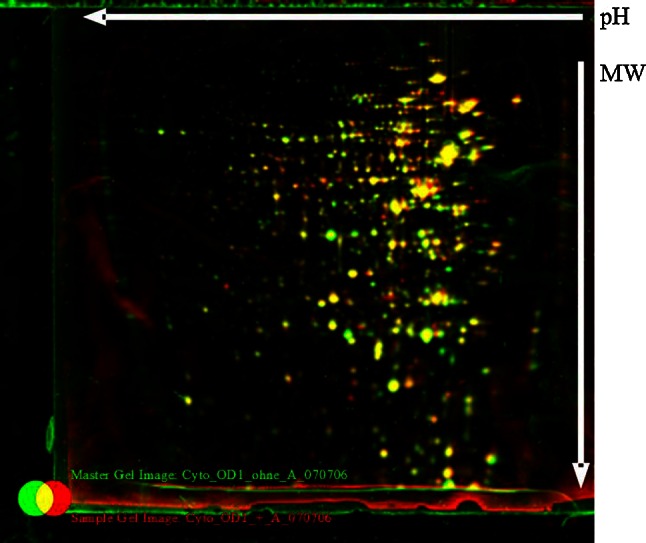
Comparison of the cytoplasmic protein pattern of *S. aureus* SH1000, with or without C-6-H, in exponential phase. Original staining and false-colour dual-channel images of 2D gels of cytoplasmic proteins without C-6-H (*green*) and with C-6-H (*red*). Proteins (200 µg) were isolated from the supernatant of SH1000 or grown in TSB^−Fe^ medium to OD_600_ 0.5, C-6-H was then added, and cultures were further incubated until OD_600_ 1.0. *Yellow* protein spots represent equal amounts in both cultures, the *green* protein spots represent higher amounts in the culture without C-6-H, and protein spots that are *red* are present in higher amounts in the presence of C-6-H

**Fig. 8:**
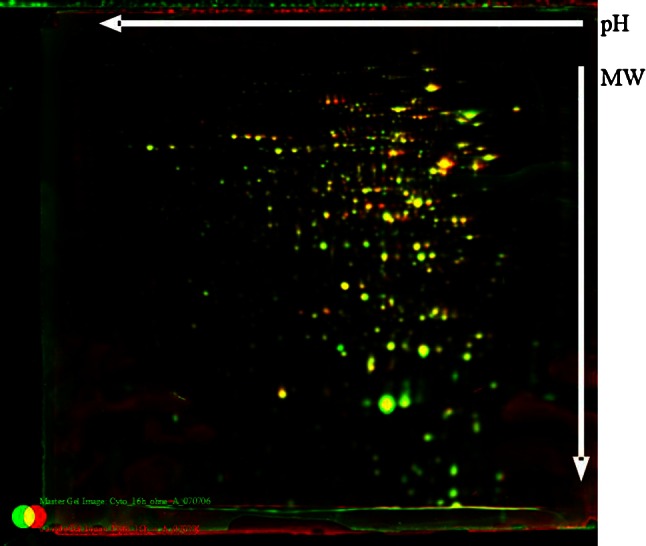
Comparison of the cytoplasmic protein pattern of *S. aureus* SH1000, with or without C-6-H, in stationary phase. Original staining and false-colour dual-channel images of 2D gels of cytoplasmic proteins without C-6-H (*green*) and with C-6-H (*red*). Proteins (200 µg) were isolated from the supernatant of SH1000 grown in TSB^−Fe^ medium to OD_600_ 0.5, C-6-H was then added, and cultures were further incubated for 16 h (stationary phase). *Yellow* protein spots represent equal amounts in both cultures, the *green* protein spots represent higher amounts in the culture without C-6-H, and protein spots that are *red* are present in higher amounts in the presence of C-6-H

One hundred and sixty-six cytoplasmic soluble proteins were identified in total and analysed by MALDI-TOF mass spectrometry. The differences in protein expression of exponential and stationary phase cell growth are shown in Table 7, and the effects of C-6-H are shown in Table 8 and Figs. 7, 8. The cytoplasmic proteins of *S. aureus* in early exponential phase (OD_600_ 1.0) and stationary phase (16 h incubation) were compared. Twenty proteins show an increase and 22 a decrease in level in stationary phase (Table 7), with a variety of predicted roles.

**Table 7:** Growth phase-dependent changes in cytoplasmic protein profile

ORF N315	Protein	Gene product	OD 1.0 versus 16 h
SA0149	CapF	Capsular polysaccharide synthesis enzyme Cap5F	2.18
SA0218	MB	Formate acetyltransferase	3.20
SA0224		Hypothetical protein, similar to 3-hydroxyacyl-CoA dehydrogenase	28.28
SA0372		Hypothetical protein	4.24
SA0506	Tuf	Elongation factor Tu	0.28
SA0506	Tuf	Elongation factor Tu	0.10
SA0513		Hypothetical protein	0.35
SA0564	ArgS	Arginyl-tRNA synthetase	0.50
SA0707		Hypothetical protein	3.06
SA0730	Pgm	Phosphoglyceromutase	0.30
SA0755		Organic hydroperoxide resistance protein-like	2.34
SA0774		Hypothetical protein	0.34
SA0793	DltA	d-alanine-poly(phosphoribitol) ligase subunit 1	0.40
SA0842	FabH	FabH, 3-oxoacyl-(acyl carrier protein) synthase homologue	0.40
SA0843	Fab	3-oxoacyl-synthase	0.44
SA0869	FabI	Enoyl-(acyl carrier protein) reductase	0.35
SA0959		GTP-binding elongation factor homologue	0.32
SA1019		Hypothetical protein	2.19
SA1045	PyrAA	Carbamoyl phosphate synthase small subunit	0.39
SA1073	FabD	Malonyl CoA-acyl carrier protein transacylase	0.48
SA1096	ClpQ	ATP-dependent protease peptidase subunit	2.46
SA1115	RibC	Riboflavin kinase/FAD synthase ribC	0.17
SA1224		ABC transporter (ATP-binding protein) homologue	0.30
SA1224		ABC transporter (ATP-binding protein) homologue	0.36
SA1307	EngA	GTP-binding protein engA	0.34
SA1309	Cmk	Cytidylate kinase	0.36
SA1343		Hypothetical protein, similar to tripeptidase	7.03
SA1410	GrpE	Heat shock protein GrpE	0.46
SA1456	AspS	Aspartyl-tRNA synthetase	0.49
SA1456	AspS	Aspartyl-tRNA synthetase	0.41
SA1522	AccA	Acetyl-CoA carboxylase carboxyltransferase subunit alpha	0.45
SA1553	Fhs	Formate-tetrahydrofolate ligase	2.77
SA1553	Fhs	Formate-tetrahydrofolate ligase	2.13
SA1609	PckA	Phosphoenolpyruvate carboxykinase	6.03
SA1609	PckA	Phosphoenolpyruvate carboxykinase	3.67
SA1609	PckA	Phosphoenolpyruvate carboxykinase	6.65
SA1692		Hypothetical protein	2.37
SA1709		Ferritin	4.20
SA1724	PurB	Adenylosuccinate lyase	2.12
SA1840		Hypothetical protein	2.02
SA1929	PyrG	CTP synthase	0.43
SA1936	LuxS	S-ribosylhomocysteinase	0.39
SA1984	Asp23	Alkaline shock protein 23	13.54
SA1984	Asp23	Alkaline shock protein 23	10.13
SA1984	Asp23	Alkaline shock protein 23	8.04
SA2098		Putative 2-hydroxyacid dehydrogenase SA2098	2.25
SA2125		Formimidoylglutamase	2.12
SA2240		Hypothetical protein, similar to para-nitrobenzyl esterase chain A	8.60
SA2317		Hypothetical protein	0.44
SA2336	ClpL	ATP-dependent Clp proteinase chain clpL	2.57

Comparison of the pattern of cytoplasmic protein expression in exponential phase (OD_600_ 1.0) and stationary phase of *S. aureus*. Proteins with a spot vol. ratio of ≥2 and ≤0.5 are shown. All proteins had a significant level of 0.05 or less (*T* test 5 % cut-off)

**Table 8:** Effect of C-6-H on cytoplasmic protein profile

ORF N315	Protein	Gene product	Expression C6H	
			OD_600_ 1.0	16 h
SA0165		Hypothetical protein, similar to alpha-helical coiled-coil	–	0.15
SA0367		NADPH-dependent oxidoreductase	–	2.13
SA0419	MetB	Cystathionine gamma-synthase	2.11	–
SA0506	Tuf	Elongation factor Tu	2.31	–
SA0506	Tuf	Elongation factor Tu	–	2.46
SA0513		Hypothetical protein	0.48	–
SA0707		Hypothetical protein	0.44	–
SA0758		Hypothetical protein, similar to thioredoxin	0.50	–
SA0869	FabI	Enoyl-(acyl carrier protein) reductase	0.40	–
SA0884		Lipoate-protein ligase homologue	–	2.09
SA1045	PyrAA	Carbamoyl phosphate synthase small subunit	0.35	–
SA1112	InfB	Translation initiation factor IF-2	–	3.52
SA1115	RibC	Riboflavin kinase/FAD synthase ribC	0.21	–
SA1258		Hypothetical protein	0.10	–
SA1522	AccA	Acetyl-CoA carboxylase carboxyltransferase subunit alpha	0.35	–
SA1868		Hypothetical protein	0.23	–
SA1943		Hypothetical protein	0.19	–
SA1959	GlmS	Glucosamine-fructose-6-phosphate aminotransferase	–	2.15
SA1959	GlmS	Glucosamine-fructose-6-phosphate aminotransferase	–	2.95
SA1959	GlmS	Glucosamine-fructose-6-phosphate aminotransferase	2.15	–
SA1984	Asp23	Alkaline shock protein 23	–	0.37
SA2084	UreC	Urease subunit alpha	14.42	–
SA2085	UreE	Urease accessory protein UreE	5.85	–
SA2085	UreE	Urease accessory protein UreE	–	3.28
SA2098		Putative 2-hydroxyacid dehydrogenase SA2098	–	2.09
SA2311		Putative NAD(P)H nitroreductase SA2311	–	2.62
SA2312	Ddh	d-lactate dehydrogenase	2.44	–
SA2336	ClpL	ATP-dependent Clp proteinase chain clpL	–	2.26
SA2336	ClpL	ATP-dependent Clp proteinase chain clpL	–	2.62
SA2400	Mqo2	Malate: quinone oxidoreductase	0.44	–
SA2400	Mqo2	Malate: quinone oxidoreductase	0.27	–

Comparison of cytoplasmic protein expression in exponential phase (OD_600_ 1.0) and stationary phase in the presence of sub-MIC C-6-H. Data for proteins with a spot vol. ratio of ≥2 and ≤0.5 are shown. All proteins had a significance level of 0.05 or less (*T* test 5 % cut-off)

In early exponential phase, the expression levels of 17 proteins were altered (≥2-fold) in the presence of C-6-H (Table 8). Eleven proteins showed a decrease and 6 an increase in level due to C-6-H. In stationary phase, 9 proteins were increased and 2 reduced in the presence of C-6-H. The greatest induction by C-6-H was seen for UreC in exponential phase (14-fold). Also, UreE showed a fivefold induction in protein level (Table 8). The increased urease level is in accordance with the array data (Table S1). A variety of other metabolic proteins were also affected by C-6-H, which alludes to a generalised effect of the inhibitor on cellular processes.

## Conclusions

There was broad (but not exact) correlation between the effects of C-6-H on gene expression and protein level revealing a pleiotropic alteration in cellular physiology and virulence. Despite multiple changes in gene expression as a result of C-6-H exposure, no single resistance mechanism could be identified, which might suggest the contribution of several factors. Our data, however, support the hypothesis that the key regulator of virulence determinant production, SaeR, is affected by C-6-H and results in the reduced expression of several toxins. This would make sense as skin fatty acids are key markers for an environment in which *S. aureus* will colonise as part of the commensal flora. Expression of components able to disrupt the host will destroy this niche and potentiate other defences, thus placing the organism at risk.

## Electronic supplementary material

Below is the link to the electronic supplementary material.

Supplementary material 1 (DOC 176 kb)
