# Supplementary material for: The effect of skin fatty acids on Staphylococcus aureus
Source: Arch Microbiol. 2014 Oct 18;197(2):245–67. doi: 10.1007/s00203-014-1048-1 (PMC4326651; doi:10.1007/s00203-014-1048-1)
Supplement: Supplementary file 1 — Supplementary material 1 (DOC 176 kb) [file 203_2014_1048_MOESM1_ESM.doc]

**Supplement S1. Effect of C-6-H on expression of genes**

The expression of genes altered after incubation with C-6-H for 10 or 60 min. Data for genes with a spot vol. ratio of ≥ 2 and ≤ 0.5 are shown. All genes had a significant level of 0.05 or less.
